# ACVR2B/Fc counteracts chemotherapy-induced loss of muscle and bone mass

**DOI:** 10.1038/s41598-017-15040-1

**Published:** 2017-10-31

**Authors:** Rafael Barreto, Yukiko Kitase, Tsutomu Matsumoto, Fabrizio Pin, Kyra C. Colston, Katherine E. Couch, Thomas M. O’Connell, Marion E. Couch, Lynda F. Bonewald, Andrea Bonetto

**Affiliations:** 1 Department of Surgery, Indiana University School of Medicine, Indianapolis, IN 46202 USA; 2 Department of Anatomy and Cell Biology, Indiana University School of Medicine, Indianapolis, IN 46202 USA; 3 Indiana Center for Musculoskeletal Health, Indiana University School of Medicine, Indianapolis, IN 46202 USA; 4 Indianapolis Project STEM, Indiana University School of Medicine, Indianapolis, IN 46202 USA; 5 Department of Otolaryngology - Head and Neck Surgery, Indiana University School of Medicine, Indianapolis, IN 46202 USA; 6 Simon Cancer Center, Indiana University School of Medicine, Indianapolis, IN 46202 USA; 7 IUPUI Center for Cachexia Research Innovation and Therapy, Indiana University School of Medicine, Indianapolis, IN 46202 USA

## Abstract

Chemotherapy promotes the development of cachexia, a debilitating condition characterized by muscle and fat loss. ACVR2B/Fc, an inhibitor of the Activin Receptor 2B signaling, has been shown to preserve muscle mass and prolong survival in tumor hosts, and to increase bone mass in models of osteogenesis imperfecta and muscular dystrophy. We compared the effects of ACVR2B/Fc on muscle and bone mass in mice exposed to Folfiri. In addition to impairing muscle mass and function, Folfiri had severe negative effects on bone, as shown by reduced trabecular bone volume fraction (BV/TV), thickness (Tb.Th), number (Tb.N), connectivity density (Conn.Dn), and by increased separation (Tb.Sp) in trabecular bone of the femur and vertebra. ACVR2B/Fc prevented the loss of muscle mass and strength, and the loss of trabecular bone in femurs and vertebrae following Folfiri administration. Neither Folfiri nor ACVR2B/Fc had effects on femoral cortical bone, as shown by unchanged cortical bone volume fraction (Ct.BV/TV), thickness (Ct.Th) and porosity. Our results suggest that Folfiri is responsible for concomitant muscle and bone degeneration, and that ACVR2B/Fc prevents these derangements. Future studies are required to determine if the same protective effects are observed in combination with other anticancer regimens or in the presence of cancer.

## Introduction

It is estimated that 1.7 million new cases of cancer will be diagnosed in the United States this year, and up to 600,000 people will die from the disease^1^. Up to 80% of all advanced cancer patients will present with signs of cachexia. This condition is characterized by depletion of skeletal muscle mass and adipose tissue, persistent muscle fatigue, anorexia, increased inflammatory state, worsened quality of life and overall shorter survival^2–6^. Experimental evidence suggests that bone loss, a disorder that primarily occurs concurrent to the formation of distal metastases in lung carcinomas, prostate and breast cancer^7,8^, is also a primary feature accompanying the development of a severe cachectic phenotype. This phenotype has been described in myeloma, colorectal and pancreatic cancers and burn-induced cachexia^9–13^.

Notably, musculoskeletal degenerations, often culminating with extensive muscle and bone loss, are the most common and most distressing symptoms associated with the development of cancer, as well as with its management and treatment^7,14–16^. We recently showed that anti-cancer regimens routinely used for the therapy of solid tumors, such as Folfiri (a combination of 5-fluorouracil, leucovorin and irinotecan), contribute to the development of cachexia and promote the loss of muscle tissue and muscle strength^17^. Similarly, chemo- and radiotherapy have been reported to play a role in bone loss by directly targeting bone mass^18,19^, promoting indirect systemic effects^20,21^, affecting bone remodeling^22,23^ or causing myelosuppression^24,25^.

The identification of muscle- and bone-derived factors for the generation of new treatment strategies is far from being accomplished. No effective therapy is currently approved for the concurrent treatment of muscle wasting and osteoporosis associated with cancer growth or anti-cancer therapies. It is estimated that cachexia ultimately accounts for 25–30% of all cancer-related deaths each year, thereby representing an important clinical problem^4,26^. While no therapies are available for the treatment of cancer-associated muscle wasting, only a few compounds, *e*.*g*. bisphosphonates and denosumab, are approved for the prevention of bone loss in combination with cancer or chemotherapy, although issues related to limited efficacy and long-term side effects have been reported in some cases^27^. Similarly, other compounds, such as the parathyroid hormone (PTH) and PTH-related protein (PTHrP) peptides, are currently being tested, while their limited applicability in association with cancer still needs to be clarified^28–30^.

In an attempt to isolate novel compounds endowed with muscle- and bone-protective properties, inhibition of the activin receptor 2B (ACVR2B) signaling was shown to enhance muscle mass, increase overall survival in experimental cancer cachexia^31,32^, and to ameliorate the dystrophic phenotype in *mdx* mice^33^. We and others showed that ACVR2B/Fc, a soluble ACVR2B fusion protein and inhibitor of receptor downstream signaling^34^, potently counteracts the Folfiri-associated myofiber atrophy in C2C12 cultures^17^ and prevents muscle wasting in combination with doxorubicin^35^ or cisplatin^36^. Administration of ACVR2B/Fc was also shown to concurrently prevent bone loss in a mouse model of osteogenesis imperfecta^37^ and in dystrophic mice^38^.

We hypothesized that inhibition of the activin signaling by means of ACVR2B/Fc could represent a novel strategy to treat musculoskeletal conditions associated with chemotherapy treatments, thereby improving both muscle and bone properties. To test our hypothesis, we treated CD2F1 mice with Folfiri, ACVR2B/Fc, or a combination of both for up to five weeks, and the effects on muscle size and strength were evaluated. In addition to muscle mass and function, we assessed the effects on bone mass, specifically trabecular bone volume fraction (BV/TV), thickness (Tb.Th), number (Tb.N), connectivity density (Conn.Dn), and trabecular separation (Tb.Sp) in trabecular bone of the femur and vertebra. In order to better understand the consequence of ACVR2B/Fc administration to healthy animals and mice treated with Folfiri, we also assessed the circulating levels of IL-6 and activin A, previously reported to play a major role in promoting cachexia and in the regulation of muscle and bone mass.

## Results

### ACVR2B/Fc preserves body weight in animals exposed to Folfiri

**Figure 1:**
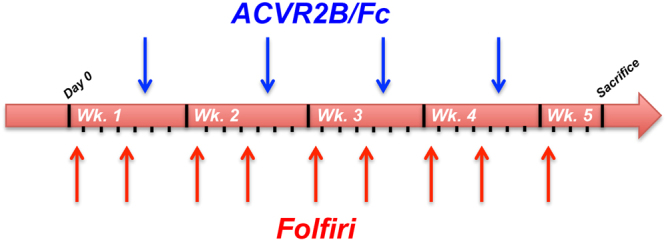
Experimental plan. Schematic representation of the experimental model and dosing schedules. The black ticks indicate the days, the blue arrows the day of ACVR2B/Fc treatment and the red arrows the day of Folfiri treatment. Wk. = week.

**Figure 2:**
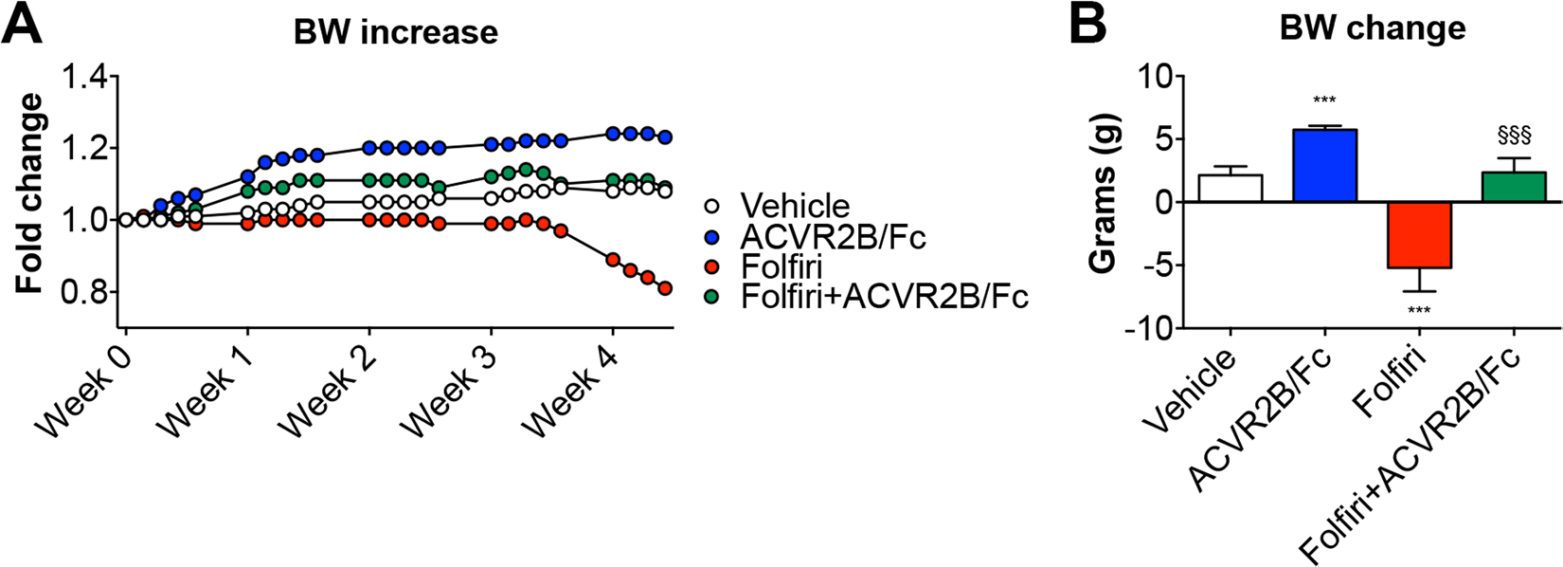
ACVR2B/Fc prevents chemotherapy-induced body weight loss. Body weight curves (**A)** and body weight change (*i*.*e*. body weight at time of sacrifice *vs*. initial body weight) **(B)** in mice exposed to Folfiri and ACVR2B/Fc for up to 5 weeks (n = 6–7). Data expressed as means ± SD. Significance of the differences: ***p < 0.001 *vs*. Vehicle; ^§§§^p < 0.001 *vs*. Folfiri.

**Figure 3:**
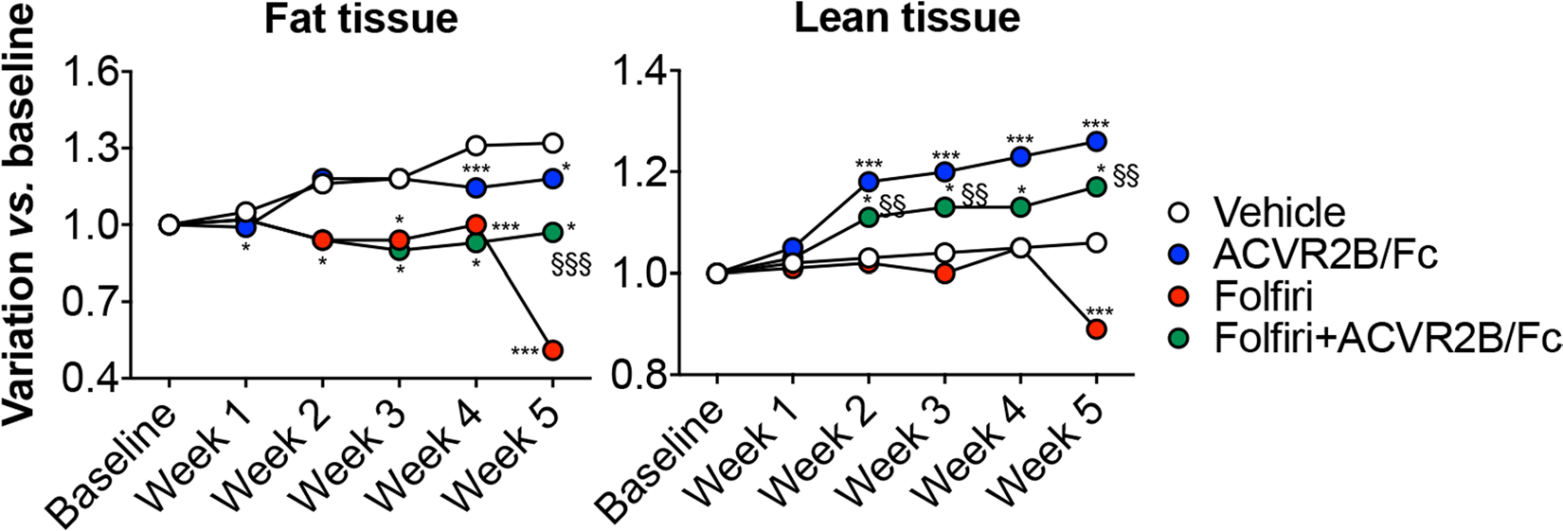
ACVR2B/Fc administration abolishes muscle depletion and partially protects against fat loss in mice treated with Folfiri. Body composition assessment in animals exposed to Folfiri and ACVR2B/Fc (n = 6–7) was performed by means of EchoMRI. Data (grams of fat or lean tissue) are expressed as fold change variation *vs*. baseline (day 0) over 5 weeks. Significance of the differences: *p < 0.05, **p < 0.01, ***p < 0.001 *vs*. Vehicle; ^§§^p < 0.01, ^§§§^p < 0.001 *vs*. Folfiri.

In order to test the use of ACVR2B/Fc for the prevention of chemotherapy-derived loss of muscle mass and muscle strength, we took advantage of a previously characterized experimental model^17^. CD2F1 male mice (8 week-old) received Folfiri (twice/weekly, i.p.), with or without ACVR2B/Fc (once/weekly, i.p.), for up to 5 weeks **(**Fig. 1 **)**. Consistent with our previous data^17,39^, after an initial lag phase, the animals that were treated with Folfiri lost about 20% of their body weight (−5.21 ± 0.32 g; p < 0.001 *vs*. Vehicle). The animals receiving the combination of Folfiri + ACVR2B/Fc displayed a substantial preservation of body mass (2.36 ± 1.14 g; p < 0.001 *vs*. Folfiri) when compared to the Vehicle-treated animals (2.13 ± 0.71 g) **(**Fig. 2A,B **)**. The animals administered the ACVR2B/Fc alone showed a marked increase in body weight (5.73 ± 0.32 g; p < 0.001 *vs*. Vehicle), in line with previous findings^31,34^ **(**Fig. 2A,B **)**. The assessment of the body composition performed by means of EchoMRI showed a precocious loss of fat tissue in the animals receiving Folfiri that ultimately was only partially preserved by ACVR2B/Fc treatment **(**Fig. 3 **)**. In line with the body weight data shown in Fig. 2, the administration of ACVR2B/Fc promoted general muscle hypertrophy and completely counteracted the loss of lean tissue as mediated by Folfiri treatment **(**Fig. 3 **)**.

### Administration of ACVR2B/Fc protects muscle mass in Folfiri-treated mice

**Figure 4:**
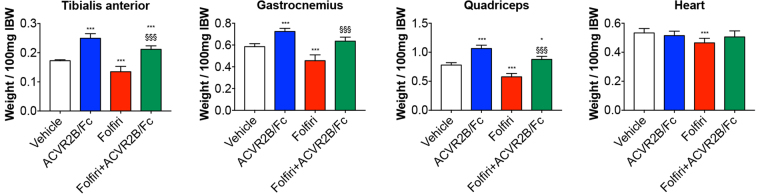
Administration of ACVR2B/Fc prevents muscle atrophy in the Folfiri-treated. Muscle weights in mice treated with Folfiri and ACVR2B/Fc for up to 5 weeks (n = 6–7). Weights were normalized to the Initial Body Weight (IBW) and expressed as weight/100 mg IBW. Data expressed as means ± SD. GSN: gastrocnemius. Significance of the differences: *p < 0.05, ***p < 0.001 *vs*. Vehicle; ^§§§^p < 0.001 *vs*. Folfiri.

**Figure 5:**
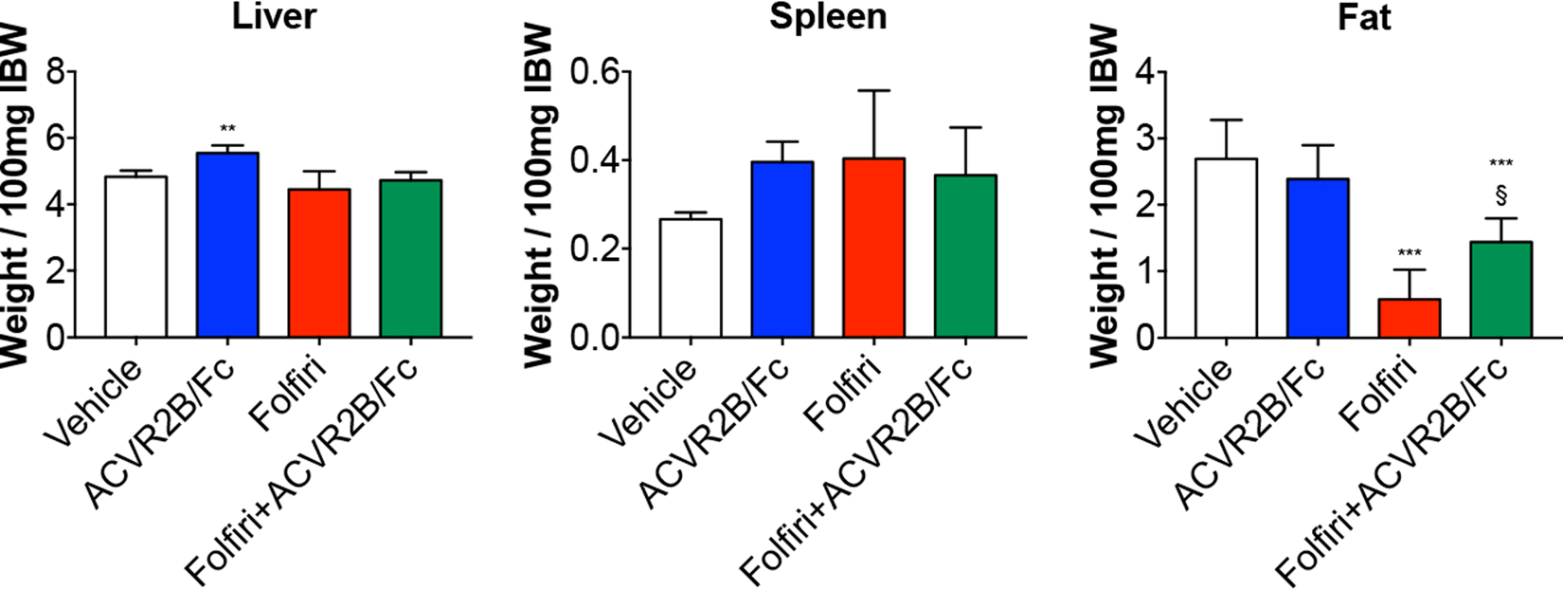
Folfiri-induced adipose tissue depletion is partially prevented by ACVR2B/Fc treatment. Liver, spleen and fat weights in mice treated with Folfiri and ACVR2B/Fc for up to 5 weeks (n = 6–7). Weights were normalized to the Initial Body Weight (IBW) and expressed as weight/100 mg IBW. Data expressed as means ± SD. Significance of the differences: **p < 0.01, ***p < 0.001 *vs*. Vehicle; ^§^p < 0.05 *vs*. Folfiri.

In line with our previous studies^17,39^, chronic administration of Folfiri to male CD2F1 mice led to marked loss of muscle tissue compared to the Vehicle-treated animals. Specifically, the chemotherapy-induced muscle wasting was consistent with markedly reduced size of tibialis anterior (−22%; p < 0.001), gastrocnemius (−22%; p < 0.001), quadriceps (−26%; p < 0.001) and heart (−13%; p < 0.001) **(**Fig. 4 **)**. To better understand whether gender-specific effects could affect the chemotherapy-associated muscle toxicity, we tested Folfiri also in female CD2F1 mice. Interestingly, the administration of the same chemotherapy regimen to female mice was promoting the occurrence of significant depletion of muscle mass (*e*.*g*. gastrocnemius: −13% *vs*. Vehicle; p < 0.05) and reduced muscle strength (−7% *vs*. Vehicle; p < 0.05) (Supplementary Figure S1). However, the chemotherapy-associated effects seemed to be less pronounced in the females, showing moderate loss of muscle mass and muscle weakness with respect to the males. Hence, we investigated the muscle-protective properties of ACVR2B/Fc in the male mice only, due to their apparently enhanced susceptibility to the effects of anti-cancer agents. ACVR2B/Fc was able to promote muscle hypertrophy, as shown by increased quadriceps size (+36% *vs*. Vehicle; p < 0.001), and to completely reverse the Folfiri-induced muscle atrophy (+13% *vs*. Folfiri; p < 0.001) **(**Fig. 4 **)**. ACVR2B/Fc treatment also caused moderate hepatomegaly (+15% *vs*. Vehicle; p < 0.01) and only partially restored the fat tissue in the Folfiri-treated mice (+53% *vs*. Folfiri; p < 0.05). No change in spleen size was recorded in any of the experimental groups **(**Fig. 5 **)**.

### ACVR2B/Fc preserves muscle size and muscle strength in animals receiving Folfiri

**Figure 6:**
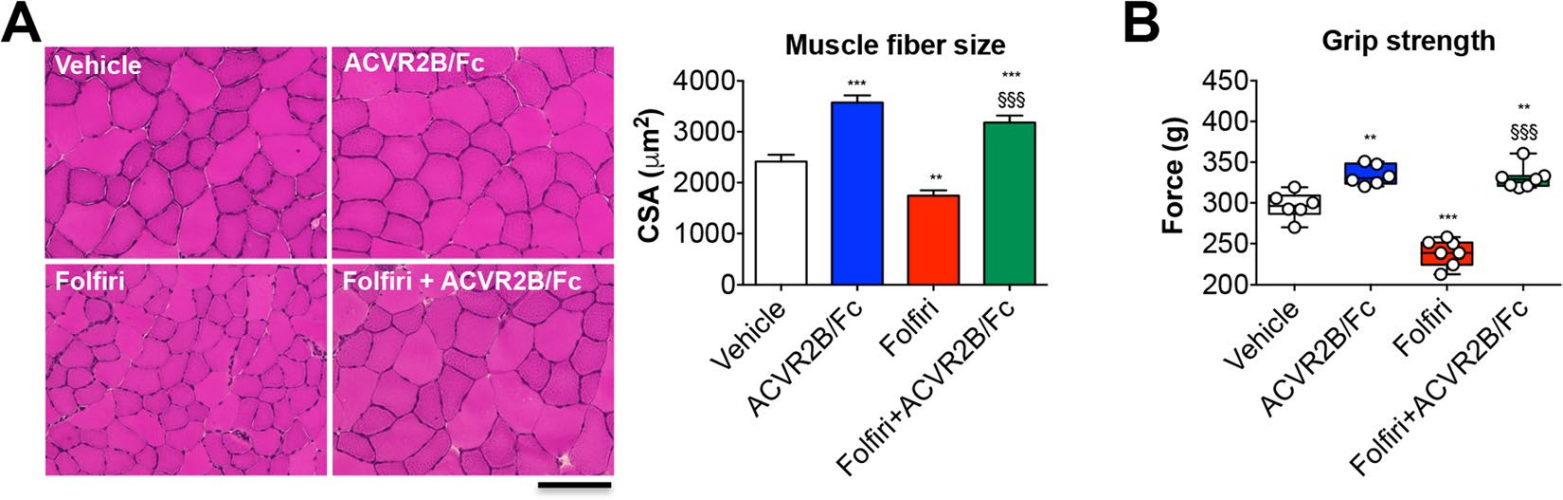
ACVR2B/Fc prevents the Folfiri-associated loss of muscle fiber size and muscle strength. Muscle morphology (H&E staining) and quantification of the cross-sectional area (CSA) in the tibialis anterior muscle of mice exposed to chemotherapy or ACVR2B/Fc. Scale bar: 100 μm. Magnification: 20X (**A)**. Whole body grip strength in animals administered chemotherapy or ACVR2B/Fc for up to 5 weeks, reported as peak force, was measured by taking advantage of a grip strength meter and expressed as the average of the three top pulls from each animal (n = 6–7) (**B)**. Data expressed as means ± SD. Significance of the differences: **p < 0.01, ***p < 0.001 *vs*. Vehicle; ^§§§^p < 0.001 *vs*. Folfiri.

Similar to Fig. 4, fiber hypertrophy was displayed in the tibialis anterior muscle following treatment with ACVR2B/Fc (+48%; p < 0.001 *vs*. Vehicle), while its administration in combination with chemotherapy presented a complete preservation of fiber size *vs*. the Folfiri-treated animals (+82%; p < 0.001) **(**Fig. 6A **)**. Weekly ACVR2B/Fc treatment, combined with chemotherapy, not only was able to fully counteract the muscle weakness associated with Folfiri (+38%; p < 0.001), but also induced a significant increase in muscle strength respect to the Vehicle-treated animals (+11%; p < 0.001) **(**Fig. 6B **)**.

### ACVR2B/Fc prevents Folfiri-induced loss of trabecular bone mass

**Figure 7:**
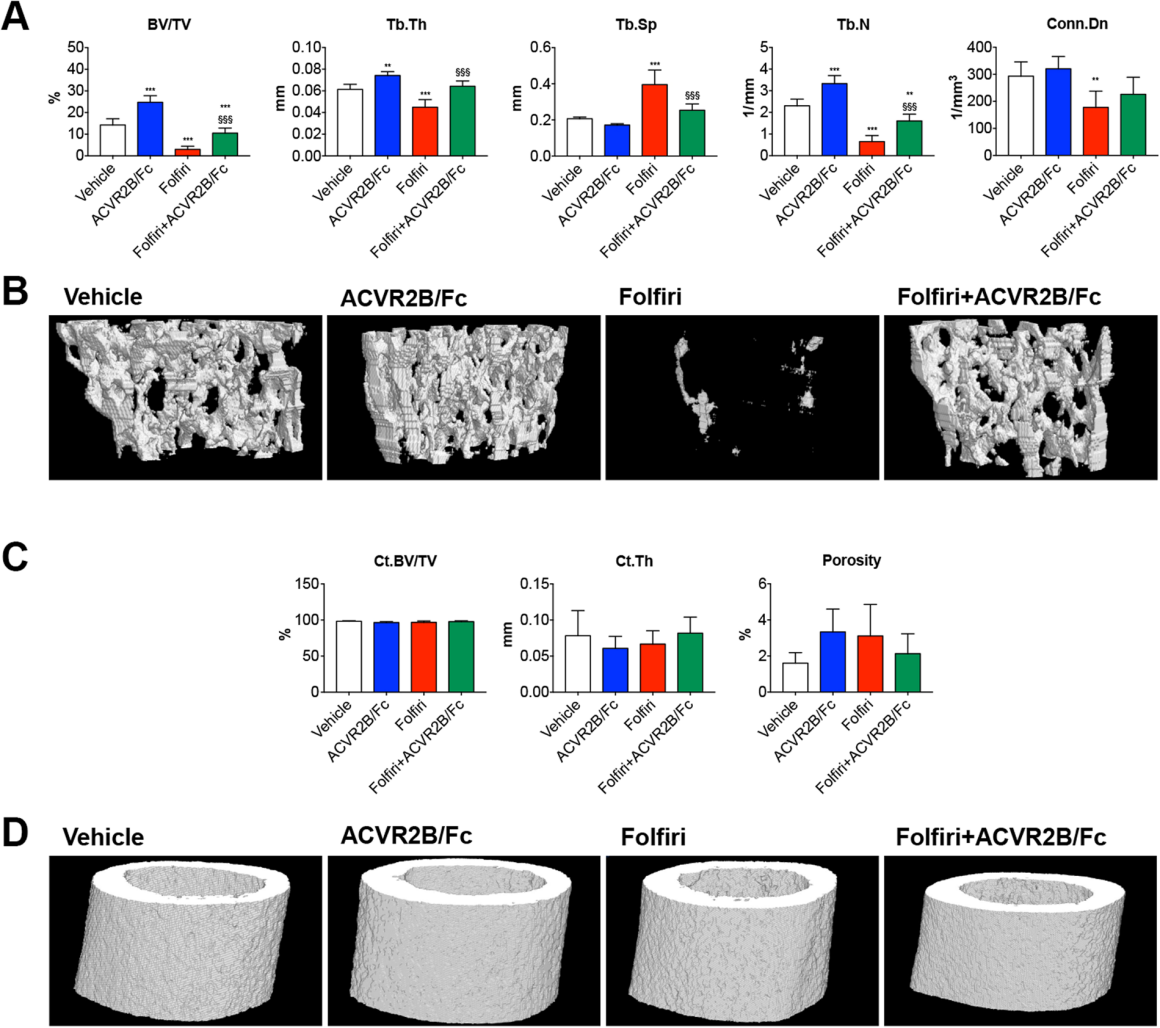
Folfiri severely affects the trabecular bone mass, but not the cortical bone, in the mouse femurs, and ACVR2B/Fc completely abolishes its effects. Quantification of bone volume fraction (BV/TV), trabecular thickness (Tb.Th), trabecular separation (Tb.Sp), trabecular number (Tb.N) and trabecular connectivity density (Conn.Dn) in the femur of mice treated with Folfiri and ACVR2B/Fc (n = 6–7) (**A**). Representative 3D rendering of μCT scan images of femur sections (**B**). Quantification of cortical bone volume fraction (Ct.BV/TV), cortical thickness (Ct.Th) and overall porosity in the femur of mice exposed to Folfiri and ACVR2B/Fc (n = 6–7) (**C**). 3D rendering of μCT scan images of cortical femur sections (**D**). Data are expressed as means ± SD. Significance of the differences: *p < 0.05, **p < 0.01, ***p < 0.001 *vs*. Vehicle; ^§§§^p < 0.001 *vs*. Folfiri.

**Figure 8:**
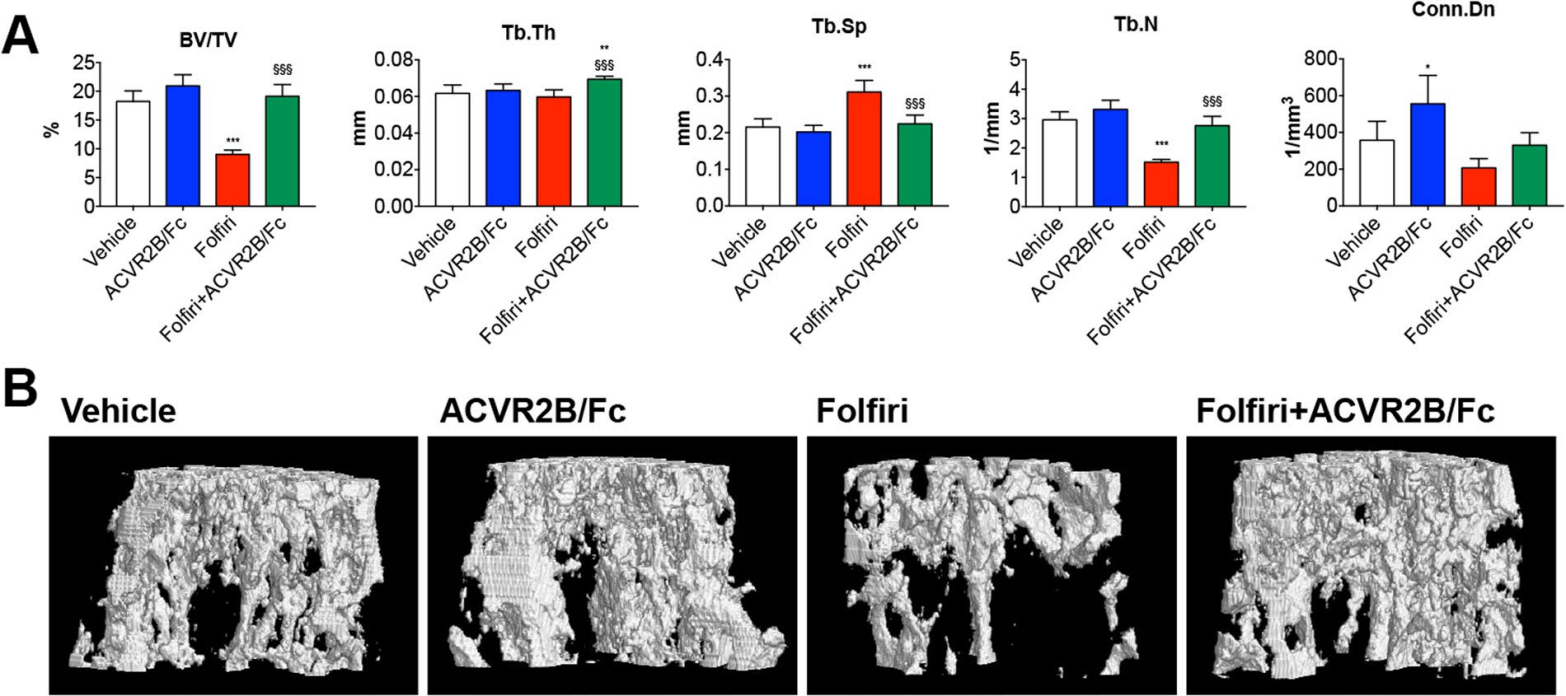
ACVR2B/Fc prevents the Folfiri-mediated effects on vertebral bone tissue. Quantification of bone volume fraction (BV/TV), trabecular thickness (Tb.Th), trabecular separation (Tb.Sp), trabecular number (Tb.N) and trabecular connectivity density (Conn.Dn) in the vertebrae of mice treated with Folfiri and ACVR2B/Fc (n = 6–7) **(A)**. Representative 3D rendering of μCT scan images of vertebral sections **(B)**. Data are expressed as means ± SD. Significance of the differences: *p < 0.05, ***p < 0.001 *vs*. Vehicle; ^§§§^p < 0.001 *vs*. Folfiri.

In order to verify whether the Folfiri-associated effects on skeletal muscle mass were reflected in a similar manner in bone, sections from femurs and vertebrae extracted from animals exposed to Folfiri and ACVR2B/Fc were analyzed by microcomputed tomography. The animals exposed to Folfiri treatment exhibited a severe loss of trabecular bone but not cortical bone in the femur **(**Fig. 7 **)**. This was evidenced by markedly reduced trabecular bone volume fraction (BV/TV; +79%; p < 0.001), thickness (Tb.Th; −26%; p < 0.001), number (Tb.N; −72%; p < 0.001) and connectivity density (Conn.Dn; −39%; p < 0.01), as well as increased trabecular separation (Tb.Sp; +91%; p < 0.001) *vs*. Vehicle **(**Fig. 7A,B **)**. The administration of ACVR2B/Fc alone not only enhanced the trabecular BV/TV (+73%; p < 0.001), Tb.Th (+21%; p < 0.01) and Tb.N (+44%; p < 0.001) *vs*. the Vehicle-treated mice, but also significantly protected the trabecular bone when combined with Folfiri, as shown by ameliorated BV/TV (+247%; p < 0.001), Tb.Th (+42%; p < 0.001), Tb.Sp (−36%; p < 0.001), Tb.N (+147%; p < 0.001) **(**Fig. 7A,B **)**. Unlike the trabecular bone, the femural cortical bone was not affected by any of the treatments, as shown by unchanged cortical bone volume fraction (Ct.BV/TV), thickness (Ct.Th) and Porosity **(**Fig. 7C,D **)**. The vertebral trabecular bone was also affected by Folfiri, as shown by reduced BV/TV (−51%; p < 0.001) and Tb.N (−49%; p < 0.001), and by increased Tb.Sp (+44%; p < 0.001) with respect to the Vehicle-treated mice **(**Fig. 8A,B **)**. The ACVR2B/Fc administration was able to counteract the Folfiri-associated effects in trabecular bone in both femur and vertebrae, thereby improving BV/TV (+112%; p < 0.001), Tb.Th (+17%; p < 0.05), Tb.Sp (−28%; p < 0.001), Tb.N (+82%; p < 0.001) **(**Fig. 8A,B **)**.

### ACVR2B/Fc reduces IL-6 and activin A in combination with chemotherapy

**Figure 9:**
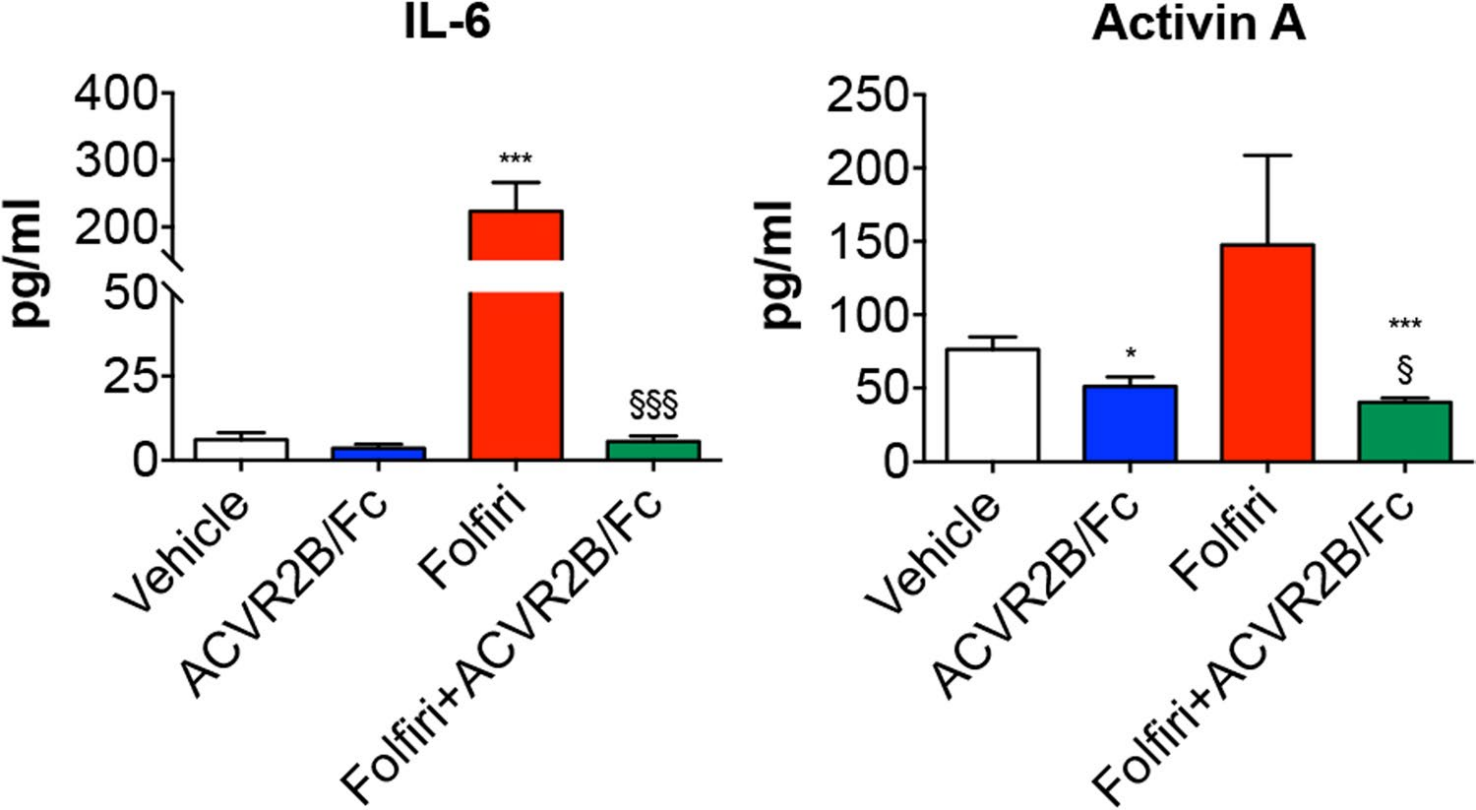
ACVR2B/Fc treatment potently reduces IL-6 and Activin A circulating levels in combination with Folfiri. Quantification of IL-6 and Activin A levels in the plasma from mice exposed to Folfiri, alone or in combination with ACVR2B/Fc (n = 6–7). Data, expressed as means ± SD, are reported in pg/ml. Significance of the differences: ***p < 0.001 *vs*. Vehicle; ^§^p < 0.05, ^§§§^p < 0.001 *vs*. Folfiri.

In order to better understand the effects associated with the administration of ACVR2B/Fc to healthy animals and mice treated with Folfiri, we assessed the circulating levels of IL-6 and activin A, previously reported to play a major role in promoting cachexia and in the regulation of muscle and bone mass^17,39^. In our animal model, the administration of Folfiri for up to 5 weeks resulted into markedly elevated IL-6 (∼36-fold increase *vs*. Vehicle; p < 0.001), which was completely prevented in combination with ACVR2B/Fc (p < 0.001 *vs*. Folfiri) **(**Fig. 9 **)**. On the other hand, despite being more elevated (+93%) following Folfiri treatment, activin A was not statistically different compared to the vehicle-treated animals. Interestingly, the combined administration of ACVR2B/Fc resulted into reduced activin A with respect to the animals receiving Folfiri (−63%; p < 0.05) or the vehicle (−30%; p < 0.05) **(**Fig. 9 **)**.

## Discussion

Cancer development often contributes to the loss of muscle mass and muscle strength, compromising the overall quality of life by decreasing both productivity and physical functioning^40–44^. Similarly, despite the recent development of novel and more effective treatment strategies capable of significantly improving life expectancy, the administration of anti-cancer therapies is known to adversely affect patients’ quality of life by producing mild-to-severe side effects. Nausea, diarrhea, anorexia and increased fatigue are the most relevant^45–47^.

We and others have reported that anti-cancer therapies cause a decline of muscle size and function (*i*.*e*. cachexia) in both animal models and cancer patients^17,47–50^. Notably, the chemotherapy-associated muscle functional deficits have been shown to positively correlate with both enhanced mortality^2,51^ and aggressiveness of chemotherapy^52,53^. Further, these complications have been reported to cause difficulty for patients to adhere to or complete treatment regimens, for delays in treatment, to cause dose limitation, and for discontinuation of therapy in cancer patients^54,55^. No remedies have been identified to relieve such conditions thus far, and the chemotherapy-associated muscle defects have been shown to persist for months to years following remission^17,56–62^.

In a similar manner, significant loss of bone mass was described in patients undergoing adjuvant chemotherapy for various gynecologic cancers^63,64^ and radiotherapy for abdominal tumors^65^. A variety of hormonal and non-hormonal compounds, frequently used in patients affected with breast and prostate cancer, was shown to promote bone resorption and bone turnover. This contributed to the development of bone loss and to the exacerbation of the overall mortality^7^. Therefore, the occurrence of bone fractures in patients with cancer or undergoing chemo-radiotherapy represents a problem of significant concern and deserves major attention.

Over the past 50 years, fluoropyrimidines, such as 5-fluorouracil (5-FU), have remained one of the most commonly used antimetabolite drugs. They have been prescribed for the therapy of metastatic colorectal cancers, resulting in significant increases in survival rates, particularly in combination with leucovorin^66^ and irinotecan, a topoisomerase I inhibitor^67^. This treatment regimen, known as Folfiri, is routinely used as a first-line chemotherapy regimen for the treatment of metastatic colorectal cancer alone, and can also be used in combination with other anti-cancer compounds, such as bevacizumab, aflibercept, cetuximab or panitumumab, to improve efficacy and response rates^68,69^.

In the present study we investigated the muscle and bone toxicities associated with the administration of Folfiri. This systemic chemotherapy regimen can have a major impact on patients’ quality of life and is often responsible for severe side effects, including myelotoxicity, gastrointestinal toxicity, enhanced morbidity and occasional mortality^70^. In line with our previous findings^17^, we report that prolonged *in vivo* Folfiri treatment accounts for severe effects on skeletal and to a lesser extent, cardiac muscles, in both male and female mice. Progressive loss of myofiber size and muscle strength occurs in animals exposed to this treatment for up to 5 weeks. Folfiri is also associated with a marked loss of adipose tissue, thereby contributing to the establishment of a general cachectic phenotype^17,39^. Interestingly, the effects described in the females were less pronounced, suggesting that gender-specific effects may play a role in determining drug toxicity. This is also in line with previous reports showing that females are likely more resistant to high-dose chemotherapy and have increased likelihood of receiving second-line anti-cancer therapies for the treatment of lung cancer^71–73^.

We also showed for the first time that chronic administration of Folfiri also associates with unexpected and severe trabecular bone loss in normal animals. Ours is not the first report to show that anti-cancer drugs may affect bone strength and quality. Indeed, Fonseca *et al*. previously reported that anti-cancer drugs, such as doxorubicin, routinely used in pediatric cancer treatment, not only promote muscle wasting^35^, but also negatively affects the overall bone morphology, as well as the femur mechanical properties in rats^74^. This is of particular importance, especially considering that patients affected with metastatic colorectal cancer usually undergo multiple rounds of chemotherapy treatment, thereby having far more chances to develop such toxicity. In addition we found that the cortical bone, however, did not seem to be significantly affected, at least within this time frame. This apparent discrepancy could be interpreted by the fact that the cortical bone, which accounts for 80% of the adult human skeleton, is typically less metabolically active than the trabecular bone^75^, and therefore likely less susceptible of the anti-proliferative and cytotoxic effects associated with the administration of chemotherapy. These effects may also be explained by the fact that 5-FU, one of the main components of the Folfiri regimen, was shown to cause marked bone loss in rats after only five consecutive days of treatment, possibly by inducing inflammation, suppressing osteoblast activity and enhancing osteoclast recruitment, therefore promoting an unbalance in bone remodeling^76^. Recent findings from Fen *et al*. showed that six cycles of 5-FU combined with cyclophosphamide and epirubicin, one of the preferred chemotherapy regimens for the treatment of breast cancer, were able to promote severe loss of trabecular bone, reduction of bone marrow cells and marrow adiposity in experimental animals^77^.

The investigation of muscle/bone crosstalk has failed to identify new targets for the treatment of chemotherapy-associated muscle wasting and osteoporosis. Unfortunately, only a few therapies, characterized by high cost and occasional long-term toxicities, are available to date. They include anti-resorptive drugs, such as the bisphosphonates, which are routinely used for the treatment and prevention of osteoporosis and bone fractures in cancer patients^78,79^. Despite this, several side effects, such as acute-phase reactions, nephrotoxicity, hypocalcemia and osteonecrosis of the jaw, have been reported^27^. On the other hand, the PTH peptides represent the only approved anabolic therapy currently in use to promote bone formation relative to bone resorption^80^, a process that primarily affects the trabecular bone tissue, whereas the effects on the cortical bone are somewhat modest and may depend on the degree of mechanical loading^81^. Safety and tolerability of these compounds, especially after prolonged administration, are still under debate, and their use in cancer patients is being evaluated. Indeed, the 2-year chronic administration of teriparatide, or rhPTH(1–34), a synthetic agonist of human PTH currently under investigation for the treatment of postmenopausal osteoporosis, was shown to potentially impact carcinogenicity by causing the formation of osteosarcomas in F344 rats, although the same finding has not been reported in the clinical setting thus far^82,83^. Moreover, despite their beneficial effects on bone mass, PTH and PTH-related protein (PTHrP) have been described to affect muscle size through the common PTH receptor (PTHR)^28–30^, therefore raising concerns that the administration of PTH peptide in patients with cancer or treated with chemotherapy, that are already at risk of significant muscle loss, may instead exacerbate their cachectic condition. Overall, it is known that muscle targeted pro-anabolic strategies can improve survival and quality of life in cancer^31,32^, as well as reduce the toxicity of anticancer drugs^17,36^. Conversely, it is not completely clear whether drugs aimed at primarily preserving bone tissue also play a role in improving the overall outcomes in the presence of a tumor. As a matter of fact, no conclusive effects of bisphosphonate administration on delayed cancer recurrence and improved survival rates have been reported so far^84^. Similarly, it is also uncertain if bisphosphonate treatment benefits muscle size and function.

In the present study we showed that inhibition of the signaling of the activin type 2B receptor represents a potent therapeutic strategy to prevent both muscle and bone loss following chemotherapy treatment. Indeed, in our study ACVR2B/Fc, a synthetic fusion peptide inhibitor of the activin receptor type 2B, was able to fully restore muscle size and function in cachectic Folfiri-treated mice. This concurs with our previous observations conducted *in vitro* using a model of chemotherapy-associated myofiber atrophy^17^. For instance, other reports suggest that the blockade of this signaling pathway mitigates the loss of muscle mass and strength in the presence of muscle atrophy due to cancer^31,32^ or its treatments^35,36^, AIDS^85^, muscular dystrophy^38,86^ or disuse^78^. ACVR2B/Fc also completely abolished the loss of bone tissue by fully preserving the trabecular bone in the animals exposed to Folfiri, as shown by the μCT analysis. Our results are consistent with previous observations reporting beneficial effects of ACVR2B/Fc treatment in mice affected with type III osteogenesis imperfecta^37^ or muscular dystrophy^38^, further showing substantial preservation of bone mass and mechanical properties. Similar to the previously published findings^79^, ACVR2B/Fc displayed bone anabolic properties, as suggested by increased trabecular bone volume fraction, thickness and number in the femurs of normal mice. Conversely, we did not observe any increase in vertebral bone parameters, with the exception of Conn.Dn, in disagreement with Bialek *et al*.^79^ Of note, no effect of ACVR2B/Fc treatment was observed in cortical bone in our model, in line with prior evidence^79^.

Overall, the abnormal activation of the signaling pathway downstream of the activin receptors is known to play a role in the regulation of both muscle and bone crosstalk, although whether the activin signaling plays a more relevant role in promoting bone abnormalities by specifically affecting either the trabecular or the cortical bone remains to be elucidated. Cancer-associated changes in the circulating levels of activin family ligands, including activin A, activin B, myostatin and GDF-11, are known to exert effects on muscle and bone homeostasis, thus contributing to the development of overt cachexia^87–89^. Similarly, Waning *et al*. suggested that the release of TGFβ due to the presence of breast cancer-associated metastatic bone lesions is responsible for muscle depletion and weakness^90^. Further, activin A, also known as a negative regulator of muscle mass and an independent prognosis factor of survival in cancer patients^88,91,92^, was described elevated in the bone matrix and was reported to play a role in the regulation of osteoclast induction and bone remodeling^93–96^. Conversely, its inhibition by means of a ligand trap soluble receptor was shown to induce bone formation and bone strength in normal and diabetic mice, as well as in ovariectomized mice affected with bone loss^97,98^. Additionally, pro-inflammatory cytokines, such as IL-1, IL-6 and TNF, known for their pro-catabolic and anti-anabolic effects in muscle tissue and for their relevant role in the pathogenesis of cachexia^99–102^, have also been shown to interact with activin A by exacerbating its detrimental effects in skeletal muscle^103,104^. In the present study we showed that IL-6 was elevated following chemotherapy treatment, and that such effect was potently counteracted by ACVR2B/Fc administration. This is in contrast with the data reported by Zhou *et al*., showing no effect of ACVR2B/Fc treatment on IL-6 levels in the presence of the C26 tumor^32^. On the other hand, circulating activin A levels did not seem to be affected by the Folfiri treatment, despite a trend towards higher concentrations that was not statistically significant, also in line with our initial observations reported in muscle tissue^17^. However, we should mention that in our work activin A was only assessed at time of sacrifice (*i*.*e*. three days after the last Folfiri injection; Fig. 1), therefore we cannot exclude that higher levels of the same factor may be detected at earlier time points or shortly after chemotherapy treatment.

Collectively, the present study shows that depletion of bone tissue occurs concurrently with severe loss of muscle mass and function in normal animals exposed to Folfiri. It also validates the blockade of the ACVR2B-dependent pathway as a potential new strategy aimed at benefitting cachectic cancer patients undergoing chemotherapy. Based on our and other’s previous observations^17,35,77,105^, the animal model used in this study represents an effective tool to study the musculoskeletal effects of anti-cancer drugs *in vivo*, a condition that must be fulfilled in order to generate meaningful animal results for human translation. Regardless, we are aware that this may also represent a limitation of our study, due to the fact that in the clinical setting only subjects affected with cancer are normally treated with chemotherapy, therefore possibly preventing us from identifying those interactions between tumor- and chemotherapy-driven mediators that may be ultimately responsible for the muscle and bone phenotypic alterations.

Furthermore, our data further support the concept that significant communication occurs in normal physiology between two closely related organs, such as muscle and bone^106^. Indeed, muscle tissue generally behaves as an endocrine organ for ‘myokines’ (*e*.*g*., IL-6, myostatin, IGF-1) playing a role in bone metabolism and function^106–109^. Conversely, bone acts as a storehouse of ‘osteokines’ (*e*.*g*., activin A, TGFβ, osteocalcin) released in physiologic and pathologic conditions, affecting the functionality of several organs, including skeletal muscle^90,110–116^. Despite all this, it is not yet known which tissue primarily influences the metabolism and function of the other, and whether the bone derangements precede the muscle effects, or vice versa. Additional efforts will be required to establish new strategies aimed at detecting early cancer-associated musculoskeletal deficits and at counteracting the simultaneous loss of muscle and bone mass following cancer treatments, especially taking into consideration that interventions primarily designed to promote muscle growth may also exert beneficial effects on bone mass, as well as bone-targeted strategies may additionally benefit skeletal muscle and its function.

## Methods

### Animals

All animal studies were approved by the Institutional Animal Care and Use Committee at Indiana University School of Medicine and were in compliance with the National Institutes of Health Guidelines for Use and care of Laboratory Animals and with the 1964 Declaration of Helsinki and its later amendments. For all the experiments described, animals were identified with a code and the investigators were blinded during allocation, animal handling, and endpoint measurements. Animals (up to 5 per cage) were housed in a pathogen-free facility at IU LARC. Male (n = 6–7) and female (n = 5) immunocompetent CD2F1 mice (Envigo, Indianapolis, IN) were used. However, after an initial characterization of the muscle-specific effects associated with Folfiri treatment alone, only the male mice were examined to investigate the beneficial effects of ACVR2B/Fc administration, in continuation with our initial study aimed at determining the effects of commonly-used chemotherapeutics on muscle mass and function^17^. More specifically, the male mice were randomized into four groups, namely Vehicle (n = 6), ACVR2B/Fc (n = 6), Folfiri (n = 7) and Folfiri + ACVR2B/Fc (n = 7). The Folfiri-treated animals received Folfiri (50 mg/kg 5-Fluorouracil, 90 mg/kg Leucovorin, 24 mg/kg Irinotecan) intraperitoneally (i.p.) twice weekly for up to 5 consecutive weeks^17^. The ACVR2B/Fc groups were administered the synthetic peptide once weekly (10 mg/kg in sterile PBS; i.p.). The Vehicle-treated animals received equal volumes of solvents only. The animals were weighed daily. At sacrifice, several tissues, including skeletal muscles, were collected, weighed, frozen in liquid nitrogen and stored at −80 °C for further studies. The tibialis anterior muscles were rapidly excised, mounted in OCT and frozen in N_2_-cooled isopentane for histology, as previously described^117^. The mouse carcasses were fixed for 2 days in 10% neutral buffered formalin, and then transferred into 70% ethanol for storage of bone and other tissues. All chemotherapy drugs were purchased from Sigma Aldrich (St. Louis, MO). ACVR2B/Fc was purified from CHO-ACVR2B/Fc conditioned medium^118^.

### Grip strength

The evaluation of the whole body strength in mice was assessed as described in^119^. Briefly, the absolute grip strength (expressed in grams) was recorded by means of grip strength meter (Columbus Instruments, Columbus, OH). Overall, 5 measurements were completed and only the top three measurements were included in the analysis. In order to avoid habituation, the animals were tested for grip strength no more than once weekly.

### Body composition assessment

Body composition, *e*.*g*. the quantification of lean (muscle) and fat (adipose) mass, was measured once weekly over the entire duration of the experiment in un-anesthetized but physically restrained mice by means of an Echo Medical systems’ EchoMRI-100 (EchoMRI, Houston, USA), as shown in^17^. This is a moderately stressful analysis, therefore the animals were assessed no more than once weekly. Data were expressed as variations over the baseline values.

### Measurement of muscle cross-sectional area (CSA)

Ten μm-thick cryosections of tibialis anterior muscles taken at the mid-belly were processed for Hematoxylin & Eosin staining. All samples were observed under an Axio Observer.Z1 motorized microscope (Zeiss, Oberchoken, Germany) and images were recorded for morphometric examination. For determination of the cross-sectional area (CSA), muscle fibers (n = 300–500 per sample) were measured by tracing the perimeter of each individual fiber using a Cintiq pen tablet input device (Wacom, Vancouver, WA, USA) and Image J 1.43 software^120^.

### Micro computed tomography (μCT) analysis of femurs and vertebrae bone morphometry

MicroCT (μCT) scanning was performed to measure morphological indices of metaphyseal regions of femurs and vertebrae, as described in^121^. After euthanasia, the mouse carcasses were fixed for 2 days in 10% neutral buffered formalin, transferred into 70% ethanol, the right femurs and the fifth lumbar vertebrae dissected, and prepared for μCT scanning on a high-throughput μCT specimen scanner. Bone samples were rotated around their long axes and images were acquired using a Bruker Skyscan 1176 (Bruker, Kontich, Belgium) with the following parameters: pixel size = 9 μm^3^; peak tube potential = 50 kV; X-ray intensity = 500 μA; 0.9° rotation step. Raw images were reconstructed using SkyScan reconstruction software (NRecon; Bruker, Kontich, Belgium) to 3-dimensional cross-sectional image data sets using a 3-dimensional cone beam algorithm. Structural indices were calculated on reconstructed images using the Skyscan CT Analyzer software (CTAn; Bruker, Kontich, Belgium). Cortical and trabecular bone were separated using a custom processing algorithm in CTAn, based on the different thicknesses of the structures. Cortical bone was analyzed by threshold 160–255 in the femoral mid-shaft. Cortical bone parameters included bone volume fraction (Ct.BV/TV), thickness (Ct.Th), and porosity. Trabecular bone was analyzed between 2.0 mm to 3.0 mm under the femoral distal growth plate and 1 mm equally between the distal and proximal growth plates in the fifth lumbar vertebra using a threshold of 80–255. Trabecular parameters included bone volume fraction (Tb.BV/TV), number (Tb.N), thickness (Tb.Th), separation (Tb.Sp), and connectivity (Conn.Dn).

### Quantification of circulating IL-6 and activin A

The circulating levels of Activin A (#DAC00B) and IL-6 (#M6000B) were measured in mouse poor-platelet plasma by using specific ELISA kits (Bio-Techne Corporation, Minneapolis, MN) and following the manufacturer’s protocol.

### Statistical analysis

Results are presented as means ± SD. Significance of the differences was determined by analysis of variance (ANOVA) followed by Tukey’s post-test. Differences were considered significant when p < 0.05.

## Electronic supplementary material

Supplementary Figure S1
