# Supplementary material for: ACVR2B/Fc counteracts chemotherapy-induced loss of muscle and bone mass
Source: Sci Rep. 2017 Oct 31;7:14470. doi: 10.1038/s41598-017-15040-1 (PMC5665981; doi:10.1038/s41598-017-15040-1)
Supplement: Supplementary file 1 — Supplementary Figure S1 [file 41598_2017_15040_MOESM1_ESM.pdf]

ACVR2B/Fc counteracts chemotherapy-induced loss of muscle and bone mass

Rafael Barreto, Yukiko Kitase, Tsutomu Matsumoto, Fabrizio Pin, Kyra C. Colston, Katherine E. Couch, Thomas M. O'Connell, Marion E. Couch, Lynda F. Bonewald, Andrea Bonetto

Supplementary Figure S1

Administration of Folfiri for up to 5 weeks determines moderate muscle loss and muscle weakness in CD2F1 female mice.

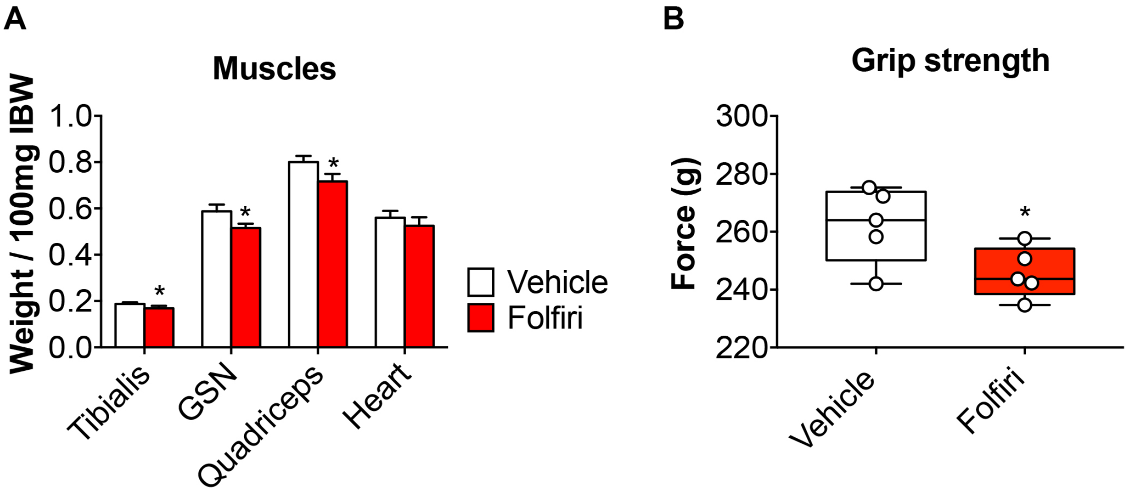

Muscle weights **(A)** and muscle strength **(B)** in female mice treated with either vehicle or Folfiri (n=5). Weights were normalized to the Initial Body Weight (IBW) and expressed as weight/100mg IBW. GSN: gastrocnemius. Data expressed as means ± SD. Significance of the differences: *p<0.05 *vs.* Vehicle.
